# Contraceptive counseling during the pandemic: practical guidelines: Number 7 - July 2021

**DOI:** 10.1055/s-0041-1735185

**Published:** 2021-08-30

**Authors:** Edson Santos Ferreira Filho, Rogério Bonassi Machado

**Affiliations:** 1 Faculdade de Medicina, Universidade de São Paulo, São Paulo, SP, Brazil; 2 Departamento de Tocoginecologia, Faculdade de Medicina de Jundiaí, Jundiaí, SP, Brazil

## Key-points:

- The access to reproductive planning and contraception services during the COVID-19 pandemic is fundamental.
- Contraceptive methods should be initiated or maintained for couples who do not wish to get pregnant during the COVID-19 pandemic.
- There is no known relationship between contraceptive methods and potential treatments against COVID-19.
- There are no specific restrictions for any contraceptive methods in women with COVID-19. Eligibility criteria must be respected for choosing the most appropriate contraceptive modality.
- Conditions must be ensured so that the consultation environment does not become a place of risk of contamination of patients and health professionals.

## Recommendations:

- Continued access to sexual and reproductive health services, especially reproductive planning and contraception is essential, even during the COVID-19 pandemic.
- Patients already using a contraceptive method and well adapted, can have their prescriptions renewed without face-to-face evaluation for another 6-12 months during the COVID-19 pandemic, respecting the eligibility criteria.
- Women using long-acting reversible contraception (LARC) may have its use extended for 1-2 years beyond the recommended in the package leaflet after counseling on risks and benefits with a health professional.
- Patients not using contraceptive who wish to prevent pregnancy during the COVID-19 pandemic should receive reproductive counseling via telemedicine and/or in person.
- Women who wish to start using LARC must have face-to-face consultation, respecting the sanitary etiquette to protect themselves and others.
- Efforts to restructure health services are essential, aiming at the tracking of asymptomatic individuals, correct diagnosis of symptomatic individuals for greater safety, and the appropriate use of personal protective equipment (PPE) by health professionals.
- There is no contraindication to the use of any contraceptive method in conjunction with COVID-19 treatments under study.
- It is acceptable, although not mandatory, to change the contraceptive method for fear of deep vein thrombosis (DVT) or pulmonary thromboembolism (PTE).

## Background

First described in China in December 2019 as an outbreak of pneumonia, COVID-19 (coronavirus disease 2019) has changed the dynamics of society and health care. Caused by SARS-CoV-2 (Severe Acute Respiratory Syndrome Coronavirus 2), 1 until 10/12/2020, this pandemic has already affected 6.7 million people in Brazil and more than 67 million people in world, resulting in 177 thousand and 1.6 million deaths, respectively. 2 To reduce the spread of the virus, people are advised to stay at home, avoid gatherings and promote social distancing, which are effective measures if properly implemented. However, they also bring dilemmas for people who need sexual and reproductive health services, including antenatal care 3 and contraception. The potential of other pandemics to significantly reduce access to family planning services is well known. 4 As reproductive counseling is a crucial strategy for reducing unintended pregnancies, 5 it is essential to reinforce its provision during the COVID-19 pandemic, respecting the health etiquette and available guidelines. 6 Reproductive choice - deciding how many children you want to have, when and if you want to have them - is one of the most fundamental human rights, in which contraception means a big step towards greater gender equality. 7

The impact of the COVID-19 pandemic on reproductive and sexual health services is clear, 8 either directly through the closure of services, or indirectly, with a reduction in manpower, financing and available equipment, because of the relocation to specific care centers for people affected by this disease. Undoubtedly, this poses significant challenges for women of reproductive age and health professionals. Given the probable increase in sexual relations in this period and greater restrictions in access to contraceptives, a substantial increase in the number of unintended pregnancies is expected - with consequent impact on public health, because it increases not only obstetric and perinatal morbidity and mortality, 7 9 but also the health system costs. 10

Brazil is already largely affected by unintended pregnancies: among 23,894 puerperal women interviewed from February 2011 to October 2012 for the “Birth in Brazil“ survey, 25.5% wanted to have waited longer and 29.9% did not want to get pregnant; only 44.6% actually wanted to become pregnant. 11 This casuistry is the result of a set of factors, including discontinuation, intrinsic failure rates of each method and inconsistent and incorrect use of different contraceptive methods. 12 An Italian cross-sectional study showed abandonment of short-term methods by half of single women, even though they maintained sporadic sexual relations that led 15% of them to an unintended pregnancy. 13 For this reason, sexual and reproductive health must be understood as a priority service, 14 especially for adolescents 15 and other more vulnerable groups.

## How should reproductive counseling be done in times of a pandemic?

The question “Can I get pregnant in a pandemic?“ has been frequent in gynecological and obstetric consultations. Data available in the current medical literature are not yet categorical in relation to greater risks in the pregnancy-puerperal cycle or for the newborn 3 and the assessment of risks and benefits requires consideration of the woman's age 16 and the planning of the couple.

If a couple does not wish to become pregnant, the choice of any modern contraceptive method, respecting the eligibility criteria, 17 18 is appropriate, 19 considering the most important features for the woman or the couple, such as efficacy, safety and availability. 20 Some methods (condoms, spermicides, diaphragms, birth control pills or emergency pills) are available over-the-counter, but not in all settings. If a woman is well adapted to her current method, she should continue to use it. Although access to all contraceptive methods can be difficult due to travel restrictions, lack of supplies and higher demand for health professionals and services, it is possible to start a new contraceptive method during the COVID-19 pandemic. The World Health Organization (WHO) also recommends the development of innovative strategies to ensure that as many people as possible have access to information and contraceptive methods during this period, including the use of telemedicine - either through conventional calls or video calls - and less restrictions on the number of bureaucratic requirements for receiving hormonal contraceptives; for example, ensuring the validity of medical prescriptions for a longer time. 21 Advice on the efficacy, adverse events and dosage of the different methods can be performed without the need for physical contact or face-to-face consultations. 22

The supply of contraceptives and menstrual and hygiene items (for example, sanitary pads, soaps, hand sanitizers) is important for women's health, women's empowerment and the full exercise of sexual and reproductive health rights, in particular for vulnerable populations. 23 In this critical period, vulnerable women are even more exposed to unintended pregnancies. This group includes women with low income and low schooling, living in rural or isolated and remote areas, prisoners, people living with the human immunodeficiency virus (HIV), cancer or other chronic diseases, as well as indigenous people, adolescents, users of illicit drugs and people with physical and/or mental disabilities. 24 In any situation, but especially in the COVID-19 pandemic, considering access to LARCs in this population should be a priority goal in the organization of public health policies. The insertion of a LARC after delivery and before hospital discharge can be a timely strategy, as it reduces the number of postpartum visits and avoids barriers to receiving contraceptive counseling. 22

For women already adapted to a contraceptive method, it is plausible to perform an additional remote prescription for the next six months for users of combined hormonal contraception (CHC) and up to 12 months for progestogen-only pills (POP). In relation to medroxyprogesterone acetate (MPA), subcutaneous formulations are interesting for self-administration. For LARC methods, including copper and levonorgestrel (LNG) intrauterine devices and subdermal etonogestrel (ENG) implants, it is appropriate to advise the patient on the possibility of prolonged use. Restrict face-to-face consultations for removal of the method if the woman wants to get pregnant or has important side effects. As long as social distancing remains, it is recommended to offer much of the contraceptive consultation process remotely. The benefits of using LARCs outweigh the risk of COVID-19 transmission. 25

In April 2020, the Specialized Contraception Commission of Febrasgo 26 issued its recommendations: 1) patients who need to start a new contraceptive method must receive face-to-face medical care guidance or, as available, by telemedicine, so that they use effective methods in addition to condoms (double protection); 2) efforts should be directed towards the continued use of methods already chosen by the woman, through active screening of users and provision of contraceptives by health agents; 3) effective actions in relation to emergency contraception can be performed by health agents or remotely (emergency contraception in Brazil is over-the-counter); 4) since the supply of LARC and the scheduling of sterilization surgery methods have been postponed during the pandemic, highly effective self-administered methods must be offered in the meantime; 5) women who use IUDs that need to be changed at the end of the conventional duration should be advised that prolonged use is an acceptable option, since studies have shown that their effectiveness can be maintained for longer than the regulated duration, usually up to 1-2 years. 26

## Is it possible to use LARC for a longer period of time, exceeding what is stated in the package insert?

There is a great deal of scientific evidence suggesting that the risk of pregnancy while using a LARC method for more than 1-2 years beyond the usual duration remains extremely low. We can advise LARC users on the effectiveness of extended use beyond the stated duration 22 and postpone IUDs and implants replacements to a time of greater control of the pandemic. In a Brazilian cohort of 228 women who had a copper IUD inserted at 25 years of age or older, pregnancy was not observed in 366 woman-years of observation among those who chose to use the device for more than 10 years. 27 Similarly, a multicenter study of 1,396 women who maintained the TCu380A IUD for 12 years showed a cumulative probability of pregnancy in 12 years of 1.9/100 women over 7,159 woman-years of observation. 28 A systematic literature review found a combined rate (11 ^th^ and 12 ^th^ year) of 0.00 pregnancies per 100 person-years. 29 Such findings allow the affirmation that advising the prolonged use of copper IUDs for women who would need to replace it during the COVID-19 pandemic is appropriate.

There are also data suggesting a possible use of the LNG IUD (52 mg, 20 μg/day) beyond five years. In a medical record review of 766 Brazilian women who maintained the same LNG IUD for more than 60 months, pregnancies were not observed during prolonged use (median of 73 months, range 61-184 months). 30 In a prospective cohort of 496 LNG IUD users, totaling a follow up of 696.9 women-year, two pregnancies were reported, that is, failure rates of 0.25 (6 ^th^ year) and 0.43 (7 ^th^ year) per 100 woman-years. 31 In a systematic review literature, was found a combined pregnancy rate of 0.02 (6 ^th^ year) and 0.03 (7 ^th^ year) per 100 woman-years. 29 In August 2020, in the United States, the Food and Drug Administration (FDA) approved the use of LNG IUD (52 mg) for six years. 32 Although in Brazil the package insert still recommends the use for five years, we believe that women can benefit from counseling on the possibility of prolonged use of this LNG IUD to avoid unnecessary exposures during the COVID-19 pandemic. Note that this guidance does not extend to the new LNG IUD (19.5 mg).

Finally, for subdermal implants, a prospective cohort study of 291 ENG implant users (mean age 29.9 years) and follow-up of 444.0 woman-years, no pregnancy was documented during the two years of use beyond the three-year approved duration. Neither there was any difference in mean serum ENG levels between groups with different body mass index (BMI): ENG levels were above the contraceptive limit of 90 pg/mL in all BMI classes at the end of three, four and five years. 31 In another study, was examined the contraceptive efficacy of the ENG subdermal implant in up to five years of use. Of the 390 participants (mean age 27.8 ± 6.1 years) who agreed to continue using the product, none became pregnant in the fourth or fifth year of observation. In this study, more than 200 women used the product for at least five years. 33 A systematic review reinforces there is sufficient evidence to recommend the prolonged use of subdermal implants. 34 Therefore, women can be advised to maintain their ENG-releasing implants for a few more months during this critical period. When advising each woman, health professionals should consider her individuality. Such recommendations are also valid for obese women.

## Are there any drug interactions between contraceptives and medications used for SARS-CoV 2 infection?

To date, there is no specific pharmacological therapy for COVID-19. The available evidence does not raise concerns about potential pharmacological interactions between contraceptive methods and possible new therapies against COVID-19. 6 The recommendation is that all health professionals ask patients about the contraceptive method in use and weigh the risks and benefits in decision making.

## What are the correlations between venous thromboembolism, hormonal contraception and COVID-19?

Despite being a frequent concern of many women and some professionals, venous thromboembolism (VTE) - whether DVT or PTE - is a rare event in women of reproductive age. The safety of combined methods is well documented, and the occurrence of contraceptive-related VTE is quite rare, especially in the young population. 35 However, the risk of VTE increases when there is an overlap of predisposing factors, that is, the combination of risk factors can lead to sufficient cumulative risk that justifies pharmacological thromboprophylaxis in users of combined methods, if hospitalized by COVID-19. 36 If, on the one hand, the Spanish guidelines aimed at women in perimenopause recommend the suspension of combined hormonal methods in cases of admission for COVID-19, 37 38 the French recommendations reinforce it does not seem reasonable to change the contraceptive method, since hemostatic changes take six to eight weeks to return to baseline. 39

We stress that women should not be encouraged to discontinue contraceptive methods unless they want to become pregnant. Given the slight increase in the risk of VTE with CHC, 40 but not with progestogen-only methods, 41 it is acceptable to advise CHC users with risk factors for VTE to change to progestogen-only methods, implants, copper IUDs or LNG IUD (if possible) for reducing the risks during COVID-19 hospitalization. However, this must be weighed against two facts: 1) discontinuation of CHC requires at least two months to restore coagulation parameters; 6 39 42 2) CHC offers significant protection against pregnancy, a condition associated with substantially higher risk of VTE. 19

## Final considerations

Given the significant risk of an increase in unintended pregnancies during the COVID-19 pandemic, we encourage women, health professionals, legislators and the whole of society to discuss sexual and reproductive health services as priority services, emphasizing contraception and the protection of women against violence. All women must have proper access to effective contraceptive methods, as well as consistent information on the topic, including LARC and emergency contraception. Women who are well adapted to their current contraceptive methods can maintain them, respecting medical eligibility criteria. Those who are not using contraceptives or need to change the current method should seek reproductive counseling. Digital technologies are useful to advise new users and answer questions. If a face-to-face consultation (for example, for physical examination in cases of unfavorable bleeding pattern or LARC insertion) is necessary, sanitary protocols must be respected, including the use of masks, temperature measurement, search for symptoms and minimization of physical contact. Current users of LARC (IUDs and implants) should be advised about the possibility of extended use, reserving face-to-face appointments for removal due to the desire to become pregnant or serious side effects. So far, limiting interactions between available contraceptive methods and medications against COVID-19 being studied are not known. Although acceptable, it is not mandatory to advise CHC users to migrate to progestogen-only methods, implants, copper or LNG IUD; considering the time until coagulation returns to baseline, adding heparin is more reasonable. Ensuring proper use of contraceptives is of utmost importance.

National Specialty Commission for Contraception of the Brazilian Federation of Gynecology and Obstetrics Associations (FEBRASGO)

President:

Rogério Bonassi Machado

Vice-President:

Ilza Maria Urbano Monteiro

Secretary:

Jaqueline Neves Lubianca

Members:

Carlos Alberto Politano

Cristina Aparecida Falbo Guazzelli

Edson Santos Ferreira Filho

Jarbas Magalhães

Luis Carlos Sakamoto

Maria Auxiliadora Budib

Mariane Nunes de Nadai

Milena Bastos Brito

Sheldon Rodrigo Botogoski

Tereza Maria Pereira Fontes

Valeria Barbosa Pontes

Zsuzsanna Ilona Katalin de Jarmy Di Bella
